# Sensitizing methicillin-resistant *Staphylococcus aureus* (MRSA) to cefuroxime: the synergic effect of bicarbonate and the wall teichoic acid inhibitor ticlopidine

**DOI:** 10.1128/aac.01627-23

**Published:** 2024-02-13

**Authors:** Selvi C. Ersoy, Richard A. Proctor, Warren E. Rose, Wessam Abdelhady, Sook-Ha Fan, Sabrina L. Madrigal, Ahmed M. Elsayed, Henry F. Chambers, Rita G. Sobral, Arnold S. Bayer

**Affiliations:** 1 The Lundquist Institute for Biomedical Innovations at Harbor-UCLA Medical Center, Torrance, California, USA; 2 Department of Medicine, University of Wisconsin School of Medicine and Public Health, Madison, Wisconsin, USA; 3 Department of Medical Microbiology and Immunology, University of Wisconsin School of Medicine and Public Health, Madison, Wisconsin, USA; 4 School of Pharmacy, University of Wisconsin-Madison, Madison, Wisconsin, USA; 5 California State University-Los Angeles, Los Angeles, California, USA; 6 University of California-San Francisco School of Medicine, San Francisco, California, USA; 7 Laboratory of Molecular Microbiology of Bacterial Pathogens, UCIBIO, Applied Molecular Biosciences Unit, Department of Life Sciences, Nova School of Science and Technology, Universidade Nova de Lisboa, Caparica, Portugal; 8 Associate Laboratory i4HB, Institute for Health and Bioeconomy, Nova School of Science and Technology, Universidade Nova de Lisboa, Caparica, Portugal; 9 David Geffen School of Medicine at UCLA, Los Angeles, California, USA; Tufts University - New England Medical Center, Boston, Massachusetts, USA

**Keywords:** methicillin-resistant *Staphylococcus aureus *(MRSA), β-lactams, NaHCO_3_-responsive, penicillin-binding proteins (PBPs), wall teichoic acid (WTA) synthesis, experimental infective endocarditis (IE)

## Abstract

Methicillin-resistant *Staphylococcus aureus* (MRSA) strains are a major challenge for clinicians due, in part, to their resistance to most β-lactams, the first-line treatment for methicillin-susceptible *S. aureus*. A phenotype termed “NaHCO_3_-responsiveness” has been identified, wherein many clinical MRSA isolates are rendered susceptible to standard-of-care β-lactams in the presence of physiologically relevant concentrations of NaHCO_3_, *in vitro* and *ex vivo*; moreover, such “NaHCO_3_-responsive” isolates can be effectively cleared by β-lactams from target tissues in experimental infective endocarditis (IE). One mechanistic impact of NaHCO_3_ exposure on NaHCO_3_-responsive MRSA is to repress WTA synthesis. This NaHCO_3_ effect mimics the phenotype of *tarO*-deficient MRSA, including sensitization to the PBP2-targeting β-lactam, cefuroxime (CFX). Herein, we further investigated the impacts of NaHCO_3_ exposure on CFX susceptibility in the presence and absence of a WTA synthesis inhibitor, ticlopidine (TCP), in a collection of clinical MRSA isolates from skin and soft tissue infections (SSTI) and bloodstream infections (BSI). NaHCO_3_ and/or TCP enhanced susceptibility to CFX *in vitro*, by both minimum inhibitor concentration (MIC) and time-kill assays, as well as in an *ex vivo* simulated endocarditis vegetations (SEV) model, in NaHCO_3_-responsive MRSA. Furthermore, in experimental IE (presumably in the presence of endogenous NaHCO_3_), pre-exposure to TCP prior to infection sensitized the NaHCO_3_-responsive MRSA strain (but not the non-responsive strain) to enhanced clearances by CFX in target tissues. These data support the notion that NaHCO_3_ is acting similarly to WTA synthesis inhibitors, and that such inhibitors have potential translational applications in the treatment of certain MRSA strains in conjunction with specific β-lactam agents.

## INTRODUCTION

Methicillin-resistant *Staphylococcus aureus* (MRSA) strains are a major clinical threat due to the large variety of invasive syndromes caused by such strains, as well as their broad antimicrobial resistances (1–3). Recently, however, a novel phenotype has been documented among large collections of clinical MRSA bloodstream infection (BSI) and skin and soft tissue infection (SSTI) isolates, termed “NaHCO_3_-responsiveness”; in this phenotype, many MRSA isolates are rendered “susceptible” *in vitro* to the β-lactams, cefazolin (CFZ) and oxacillin (OXA), in the presence of physiologically relevant concentrations of NaHCO_3_ (4–6). The translatability of this *in vitro* phenotype has been established in both *ex vivo* and *in vivo* endocarditis models (4, 7, 8).

Mechanistically, NaHCO_3_-mediated susceptibility to CFZ and OXA appears related to multiple impacts on penicillin-binding protein (PBP) 2a expression and functionality (9–11). In addition, NaHCO_3_ has been shown to affect the expression and production of components required for PBP2a activity, including the PBP2a chaperone system, *vrsA-prsA*, and wall teichoic acid (WTA) synthesis (9, 12). Of interest, disruption of WTA synthesis, with agents such as ticlopidine (TCP), tarocins, and tunicamycin, is also known to sensitize MRSA to β-lactams, especially those that specifically target PBP2 (13–16). These latter anti-WTA agents share the capacity to inhibit the activity of enzymes involved in early WTA synthesis (13, 15–17).

As mentioned above, we recently demonstrated that NaHCO_3_ was capable of altering WTA production in a small cohort of MRSA BSI isolates, and that NaHCO_3_ and TCP were similarly capable of sensitizing MRSA to the PBP2-targeting β-lactam, cefuroxime (CFX) (12). There were no further *in vitro* synergistic enhancements of NaHCO_3_ plus TCP on CFX sensitization by minimum inhibitory concentration (MIC) assay, suggesting that NaHCO_3_ and TCP may have the same biological target.

In the present study, we investigated (i) the ability of NaHCO_3_ and TCP to sensitize MRSA to CFX in BSI and SSTI isolates in both static (MIC) and dynamic (time-kill) assays; and (ii) both the *ex vivo* and *in vivo* translatability of *in vitro* susceptibility assays with CFX, NaHCO_3_, and/or TCP, utilizing *ex vivo* simulated endocarditis vegetations (SEV) and rabbit infective endocarditis (IE) models.

## RESULTS

### *In vitro* synergy of TCP, NaHCO_3_, and CFX

Previously, both TCP and NaHCO_3_ were each shown to sensitize NaHCO_3_-responsive MRSA BSI isolates to the PBP2-specific inhibitor, CFX (12). In the current cohort, which has been enlarged by the addition of MRSA SSTI isolates, a similar phenotype was observed, wherein NaHCO_3_-responsive MRSA isolates were sensitized to CFX by TCP or NaHCO_3_; in contrast, the susceptibilities of non-responsive strains were not impacted by these agents (Table 1). As observed before, the MICs of CFX were similar for NaHCO_3_-responsive strains exposed to TCP or NaHCO_3_ alone, although no additional CFX MIC reductions occurred when TCP and NaHCO_3_ were combined.

**TABLE 1:** Minimum inhibitory concentrations (MICs) of cefuroxime (CFX) for NaHCO_3_-responsive and non-responsive MRSA isolates grown in the presence and absence of TCP and/or NaHCO_3_

	Strain	Isolate source	Cefuroxime MIC (µg/mL)			
			Ca-MHB Tris		Ca-MHB Tris 44 mM NaHCO_3_	
			W/o TCP^*a*^	+ TCP^*b*^	W/o TCP	+ TCP
NaHCO_3_-responsive	MRSA 11/11^*c*^	BSI^*d*^	256	8	4	8
	MW2*^c^*	BSI	128	8	8	8
	8010	SSTI*^d^*	256	16	8	16
	8029	SSTI	256	8	8	16
	8058	SSTI	128	16	16	8
	8074	SSTI	256	32	16	32
	8129	SSTI	128	16	8	8
Non-responsive	COL^*c*^	BSI	>512	>512	512	>512
	BMC1001^*c*^	BSI	>512	>512	>512	>512
	8049	SSTI	>256	>256	>256	>256
	8080	SSTI	>256	>256	>256	>256
	8086	SSTI	32	32	32	16
	8096	SSTI	>256	>256	>256	>256
	8131	SSTI	16	16	8	16

^*a*^ TCP = ticlopidine.

^*b*^ TCP exposure is 32 µg/mL in all conditions.

^*c*^ Data previously published in Ersoy et al. (12).

^*d*^ BSI, bloodstream infection; SSTI, skin and soft tissue infection.

**Fig 1:**
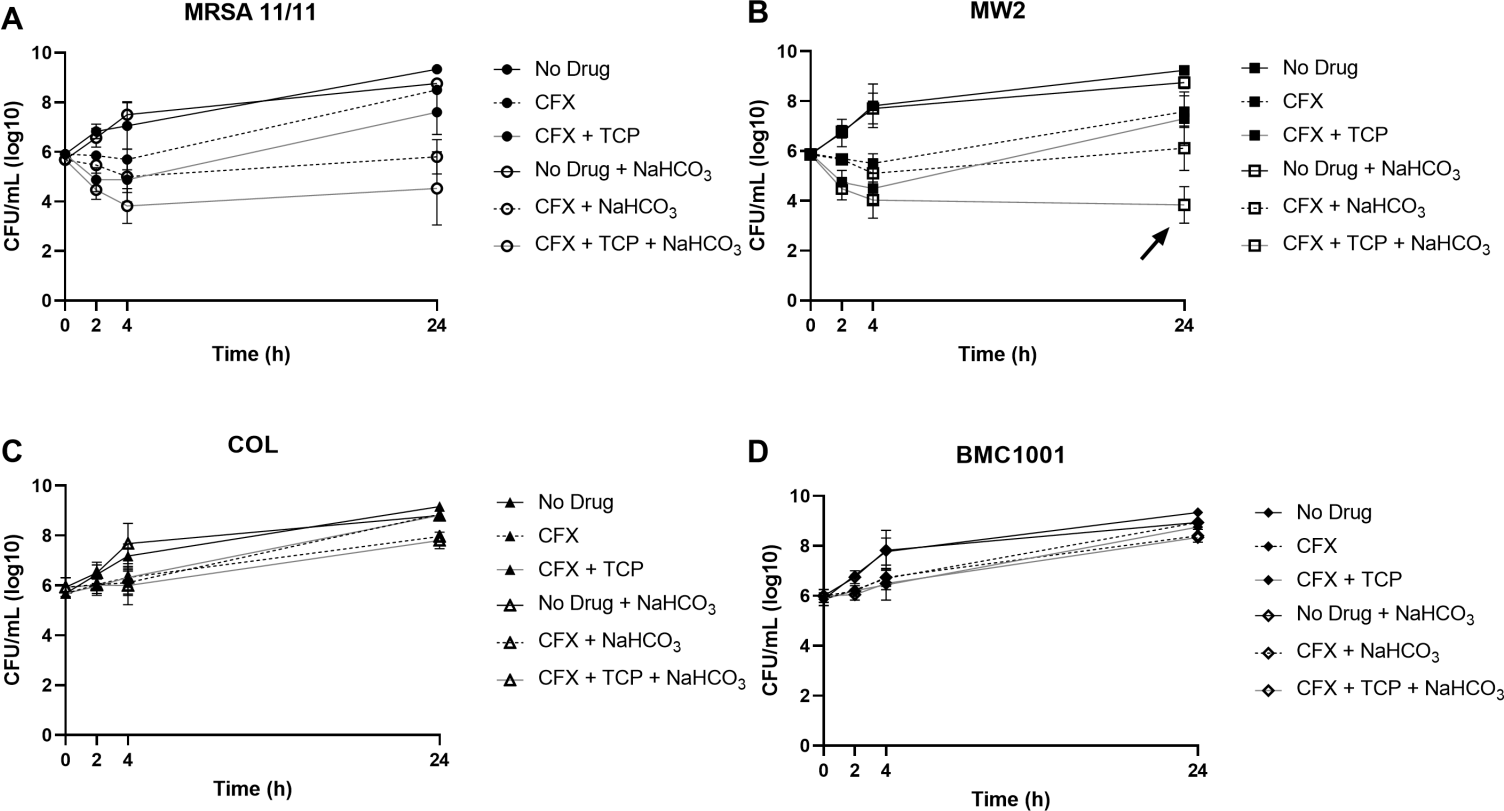
Time-kill curves for NaHCO_3_-responsive strains MRSA 11/11 (**A**) and MW2 (**B**), and non-responsive strains COL (**C**) and BMC1001 (**D**) exposed to CFX, with or without NaHCO_3_ and TCP. Cells were incubated without drug for 3 h to enter log phase. For all assays, drug concentrations are 30 µg/mL CFX and 32 µg/mL TCP.

To further quantify the dynamic impacts of TCP and NaHCO_3_ on CFX susceptibility, time-kill assays were used in four prototype strains: two NaHCO_3_-responsive (MRSA 11/11 and MW2) and two NaHCO_3_-non-responsive (COL and BMC1001). Although the MICs of CFX were similar in the presence of TCP or NaHCO_3_ alone or in combination, the combination of TCP plus NaHCO_3_ resulted in several logs of increased killing by CFX in NaHCO_3_-responsive strains at 24 h vs each agent alone, CFX and NaHCO_3_ in combination, or TCP and CFX in combination (Fig. 1A and B). This triple combination exerted “synergy” for strain MW2 (>2 log_10_ CFU/mL reductions, *P* < 0.0001 CFX + TCP + NaHCO_3_ vs both CFX + TCP or CFX + NaHCO_3_ at 24-h exposures) (Fig. 1B). This suggested the presence of synergistic bactericidal effects of CFX plus TCP plus NaHCO_3_ in combination that were not observed in more static MIC assays. As expected, TCP and NaHCO_3_, alone or in combination, did not enhance bacterial killing by CFX in non-responsive strains (Fig. 1C and D).

### *Ex vivo* CFX synergy with NaHCO_3_ and TCP in the SEV model

**Fig 2:**
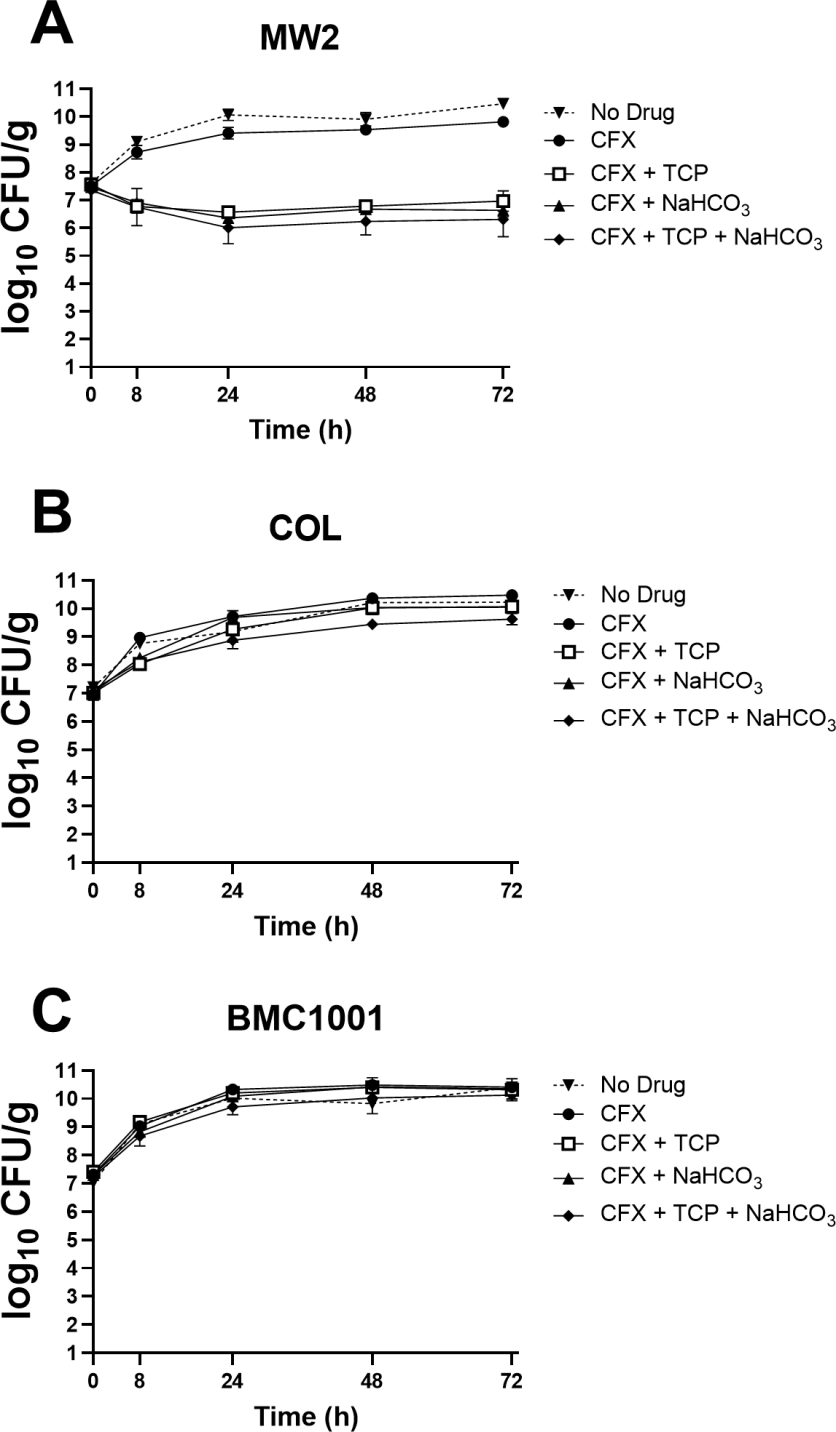
SEV kill curves for NaHCO_3_-responsive strain MW2 (**A**), and non-responsive strains COL (**B**) and BMC1001 (**C**) exposed to CFX, with or without NaHCO_3_ and TCP. CFX dosing mimics human PK/PD, while TCP dosing was optimized to reach a steady state of 8 µg/mL throughout the experiment.

This SEV model mimics two key microenvironments that MRSA strains are exposed to *in vivo*: (i) high-inoculum growth within cardiac vegetations; and (ii) human-equivalent pharmacokinetic-pharmacodynamic (PK-PD) dosing regimens. Thus, this model is widely considered as a “bridge” between *in vitro* and *in vivo* studies (18, 19). SEV time-kill assays for the NaHCO_3_-responsive strain, MW2, demonstrated (i) no difference in 72-h SEV growth curves between exposures to chamber fluid media alone (CA-MHB Tris), CFX alone or media supplemented with NaHCO_3_ or TCP alone (growth curves for supplementation with NaHCO_3_ or TCP alone not shown); and (ii) a >3 log_10_ CFU/g reduction for CFX supplemented media with TCP, NaHCO_3_, or both, indicating synergy between CFX combined with TCP and/or NaHCO_3_ (Fig. 2A), as predicted by *in vitro* MIC studies (Table 1). Neither combination regimen exerted a bactericidal effect over the 72-h exposure period. In contrast, for the non-responsive strains, COL or BMC1001, in the SEV model, neither TCP nor NaHCO_3_ enhanced killing of CFX, as expected from *in vitro* studies (Fig. 2B and C; Table 1).

### CFX synergy with endogenous NaHCO_3_ and/or TCP pre-exposures in experimental IE

**Fig 3:**
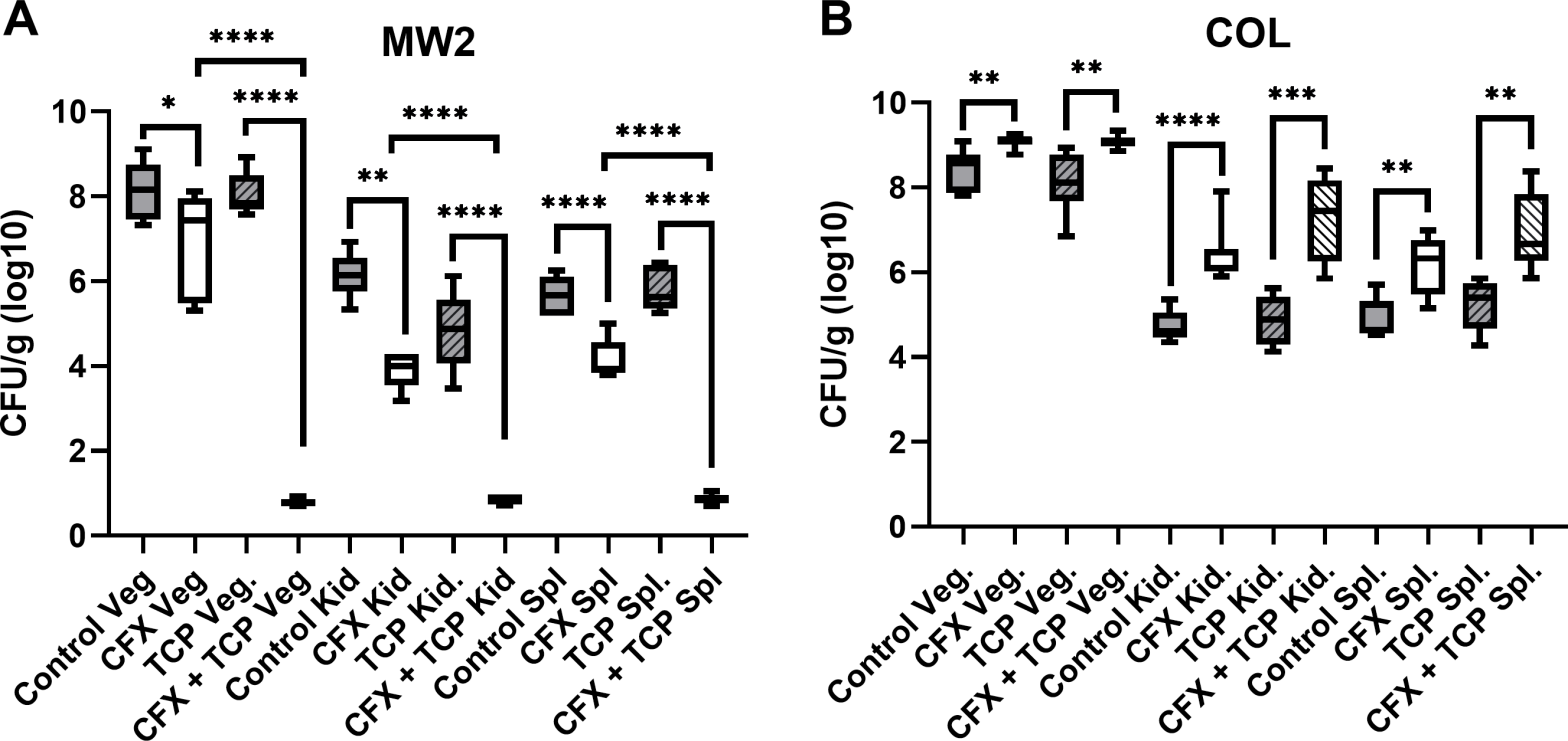
Tissue burdens (CFU/g) of NaHCO_3_-responsive strain MW2 (**A**) and non-responsive strain COL (**B**) following infection in a rabbit model of IE. Rabbits were grouped as follows: control = infected with MW2 or COL and untreated; CFX = infected with MW2 or COL and treated with CFX; TCP = infected with MW2 or COL that were exposed to TCP prior to infection and untreated; TCP + CFX = infected with MW2 or COL that were exposed to TCP prior to infection and treated with CFX. Veg. = cardiac vegetation, Kid. = kidney, Spl. = spleen. Statistics were calculated by an unpaired Student’s *t*-test, *P < 0.05, **P < 0.01, ***P < 0.001, ****P < 0.0001.

To investigate the ability of TCP to sensitize MRSA to CFX in the presence of endogenous HCO_3_, a rabbit model of experimental IE was used. MRSA cells were pre-exposed to TCP prior to infection to “sensitize” them for potential CFX synergy *in vivo*. Since TCP has potent anti-platelet aggregation activity and the pathogenesis of IE is highly platelet-dependent (15, 20–22), this pre-exposure strategy was employed rather than direct TCP treatments of animals. Furthermore, TCP is rapidly and extensively metabolized in the liver (23). As predicted by MIC, time-kill, and SEV data above, CFX treatment of rabbits infected with the “un-sensitized” NaHCO_3_-responsive strain, MW2, resulted in modest but significant reductions (0.5 to 2 log_10_ CFU/g reductions) in MRSA bio-burdens in all target tissues (Fig. 3A). Of note, when MW2 was “sensitized” by TCP exposure prior to infection, CFX treatment resulted in significantly more MRSA killing (4 to 7 log_10_ CFU/g reductions) than infection with un-sensitized cells, to near sterilization (detection-limit) levels in all three target tissues (Fig. 3A). As expected, CFX treatment of rabbits infected with the non-responsive strain COL, with or without TCP priming, did not result in any reductions of target tissue MRSA bio-burdens (Fig. 3B).

## DISCUSSION

The ability to “re-purpose” antibiotics with broad efficacy, which are relatively inexpensive and have well-known modes of action and favorable safety profiles, would be a great benefit to physicians in treating complicated MRSA infections (24, 25). Current treatment options for MRSA infections are more limited, and tend to be less effective and more toxic than standard-of-care β-lactams used for the treatment of methicillin-susceptible *S. aureus* (MSSA) (26–28).

In the current study, we demonstrated five major findings: (i) BSI and SSTI NaHCO_3_-responsive (but not NaHCO_3_-non-responsive) MRSA isolates were sensitized to growth inhibition and killing by the widely used, broad-spectrum, second-generation cephalosporin, CFX (29), in the presence of NaHCO_3_ or TCP both *in vitro* and *ex vivo*; (ii) in growth-inhibitory MIC assays, the reductions in baseline CFX MICs, when combined with either NaHCO_3_ or TCP alone, were not synergistically enhanced by combining all three agents (suggesting that NaHCO_3_ and TCP may have the same biological target); (iii) in contrast, in time-kill assays, the combination of TCP plus NaHCO_3_ did, indeed, enhance the activity of CFX against NaHCO_3_-responsive MRSA, as compared to CFX plus either TCP or NaHCO_3_; (iv) in a SEV model, exposure to CFX with TCP and/or NaHCO_3_ resulted in similar levels of synergistic growth inhibition compared to CFX exposure alone in NaHCO_3_-responsive MRSA; and (v) TCP pre-exposures sensitized NaHCO_3_-responsive MRSA to CFX-mediated target tissue MRSA clearances, in the presence of endogenous HCO_3_ in experimental IE (i.e., HCO_3_ naturally existing in blood and tissues, typically ranging from ~22 to 45 mM [4, 30]).

Of note, there were discrepancies between the various *in vitro*, *ex vivo*, and *in vivo* assays in determining whether combining CFX with both TCP and NaHCO_3_ resulted in enhanced growth inhibition compared to exposure to CFX with either TCP or NaHCO_3_ alone. Although the MIC and SEV assays did not demonstrate enhanced synergy with the triple combination (CFX + TCP + NaHCO_3_), such enhanced efficacy was observed by time-kill assay and pre-exposure to TCP in the experimental rabbit IE model, in NaHCO_3_-responsive MRSA. We attribute these discrepancies to several factors, including (i) differences in the “metabolic state” of the organism in MIC and SEV vs time-kill and *in vivo* assays, making MRSA in the latter conditions more “susceptible” to such synergy; and/or (ii) the increased production of the inhibitory target (WTA) when initially exposed to TCP and CFX during exponential-phase growth (time-kill assays). Regarding the differences in triple combination synergy in the *ex vivo* vs *in vivo* model, the latter used TCP priming, which appears to further sensitize NaHCO_3_-responsive MRSA to the effects of CFX. Furthermore, exposure to WTA synthesis-inhibiting molecules has been shown to directly reduce MRSA virulence (31), which may explain the enhanced clearance of TCP pre-exposed NaHCO_3_-responsive MRSA by CFX in the experimental IE model.

Antibiotic susceptibility is strongly tied to the growth state of the organism, which typically exhibits higher levels of “resistance” to antimicrobials during non-planktonic growth in a biofilm (vs planktonic growth in standard *in vitro* growth media) (32). These differences can confound *in vitro*-determined levels of antibiotic resistance, which typically measures resistance in a planktonic state, against those exhibited during biofilm growth as found in selected host infections. Importantly, the studies presented herein demonstrate that NaHCO_3_ and TCP sensitize MRSA to CFX in both *in vitro*, planktonic growth states (MIC and time-kill assays), as well as in the *ex vivo* SEV model, in which MRSA cells are growing deeply within platelet-fibrin clots, in a biofilm-like state similar to what is observed during infection within a human endovascular lesion in endocarditis (8, 33). These similar sensitization outcomes observed in both these models, in which MRSA growth states differ greatly, further support the notion that both NaHCO_3_ and TCP would be capable of sensitizing certain MRSA strains (i.e., NaHCO_3_-responsive strains) to CFX during actual human infection. Finally, the *in vivo* verification of the efficacy of CFX in TCP-pretreated NaHCO_3_-responsive strains (in the presence of endogenously derived NaHCO_3_) in clearing MRSA from human-like infected cardiac vegetations fully underscores the above concepts.

The WTA biosynthetic pathway is a promising target for new antimicrobials, as WTAs are involved in a wide range of key physiological functions including bacterial:host interactions, bacterial virulence, biofilm formation, and peptidoglycan functionality, including cell division and autolysin activity (34). Additionally, WTAs are essential for the cooperative action of PBP2 and PBP4 that results in highly cross-linked peptidoglycan. Both impairment of the first step of WTA synthesis, mediated by the transferase, TarO, and deletion of *tarO* result in sensitization of MRSA to many β-lactams, including those specific for PBP2, such as CFX (15). Exposure to NaHCO_3_ simulates Δ*tarO*-deletion phenotypes in NaHCO_3_-responsive strains; however, NaHCO_3_ has no direct impact on *tarO* gene expression, either transcriptionally or translationally (12). Thus, NaHCO_3_ appears to impact TarO post-translationally and reduces WTA production exclusively in NaHCO_3_-responsive MRSA (12).

**Fig 4:**
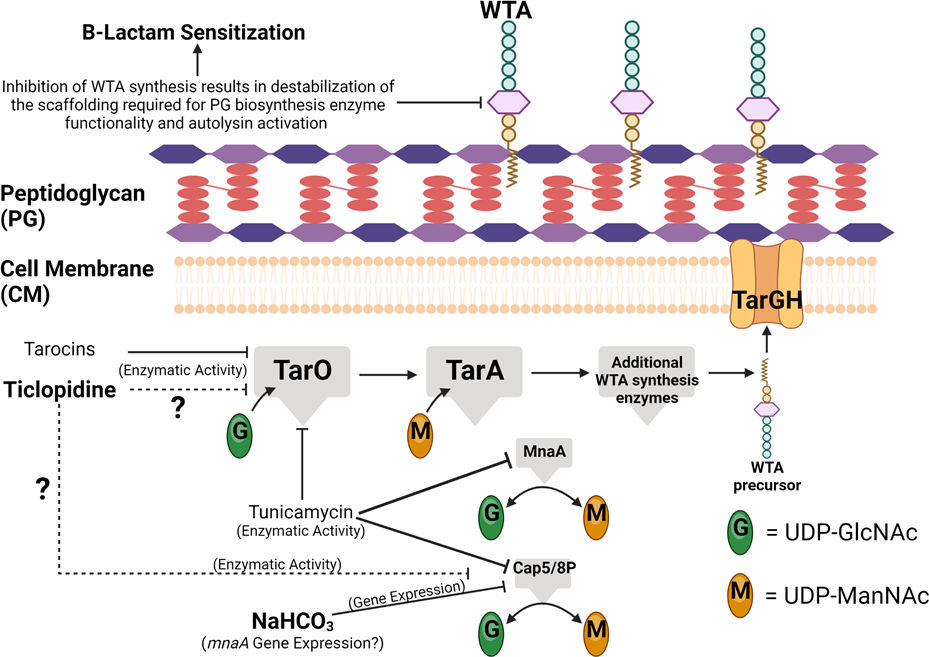
Schematic of potential impacts of TCP and NaHCO_3_ on WTA biosynthesis. Previous studies have demonstrated that TCP inhibits WTA biosynthesis (similarly to tarocins and tunicamycin), potentially via impacts on TarO functionality (13, 15, 17). NaHCO_3_ has been demonstrated to specifically inhibit the expression of *cap5/8P* in a NaHCO_3_-responsive MRSA strain (36). Inhibition of *cap5/8P* expression, coupled with inhibition of TarO enzymatic activity (via direct or indirect mechanisms), could result in disruption of WTA biosynthesis, as previously observed (12), sensitizing NaHCO_3_-responsive MRSA to β-lactams. Created with BioRender.com.

The precise anti-WTA mechanisms of TCP are not totally defined; however, like NaHCO_3_, TCP most likely targets TarO and synergizes with CFX against MRSA (15, 17). It was previously reported that TCP inhibits the enzymatic activity of TarO (15); however, it remains unclear if TCP directly binds to TarO or whether it inhibits the enzymatic activity of TarO via secondary pathways (17). One potential secondary target of TCP and NaHCO_3_ is Cap5P, the 2’-epimerase that interconverts UDP-GlcNAc and UDP-ManNAc, the substrates of TarO and another early WTA synthesis step, mediated by TarA (16, 35). Inhibition of UDP-GlcNAc and UDP-ManNAc interconversion has been shown to inhibit WTA synthesis and sensitize MRSA to β-lactams (16). Interestingly, NaHCO_3_ was previously demonstrated to inhibit the expression of *cap5/8P* specifically in NaHCO_3_-responsive strains (36), and certain *cap5P* genotypes were specific to non-responsive MRSA strains in a collection of SSTI isolates (6). Although speculative, these latter data provide one potential mechanism in which NaHCO_3_ and TCP may be inhibiting the expression and/or function of *cap5P*/Cap5P, thereby, repressing the enzymatic activity of TarO and disrupting WTA synthesis specifically in NaHCO_3_-responsive MRSA. A schematic illustrating this hypothesis is provided in Fig. 4.

Deletion of *tarO* or exposure to targocil (another WTA synthesis-inhibiting agent that targets the translocator, TarG) alters the expression of a similar cadre of virulence and peptidoglycan biosynthesis genes as observed with exposure to NaHCO_3_ in NaHCO_3_-responsive MRSA, including *pbp2*, *fmtA*, *vraX*, *sasD*, *fnbA*, and *fnbB* (14, 36, 37). This provides yet another line of evidence that NaHCO_3_ may well impact multiple steps in the WTA biosynthetic pathway. Assessing the ability of NaHCO_3_ and/or TCP to further sensitize a Δ*tarO* NaHCO_3_-responsive MRSA strain to CFX may reveal whether these compounds do, in fact, target TarO and/or act on another aspect of WTA synthesis. In addition, studies utilizing the TarG inhibitor, L-638, in combination with either NaHCO_3_ or TCP may help elucidate their combined impacts on early WTA synthesis. Inhibition of TarG by L-638 has been shown to inhibit cell growth, and suppression of this activity can be achieved by concomitant exposure to early WTA synthesis inhibitors (17).

Other known early WTA synthesis inhibitors, such as tunicamycin and tarocins A and B, have also been shown to sensitize various MRSA strains (including the NaHCO_3_-non-responsive strain, COL) to a number of β-lactams, perhaps indicating a more potent or broader spectrum of anti-WTA activity than seen with TCP and/or NaHCO_3_ (13, 17). Furthermore, tunicamycin and tarocins showed higher *in vitro* inhibitory activity against TarO than TCP, and although both drug classes act through different mechanisms on WTA synthesis, their synergic effect with β-lactams was demonstrated to be more potent than the effect of TCP (17). As only a relatively modest synergistic effect was observed between NaHCO_3_ and/or TCP with CFX *in vitro* and *ex vivo* in the present study, it would be of interest to determine whether a more robust level of synergy might be observed between NaHCO_3_, β-lactams, and either tunicamycin or tarocins *in vitro* and *ex vivo*. Indeed, the significant synergy demonstrated with TCP “sensitizing” used in the *in vivo* IE model indicates a potential role of sequential inhibition of this pathway for maximal anti-MRSA effects. Furthermore, *in vivo* studies in a rabbit IE model, similar to the present studies, utilizing selected β-lactams and tunicamycin or tarocins for the treatment of NaHCO_3_-responsive and non-responsive MRSA would be very informative. Such investigations are currently in progress in our laboratories.

One limitation of this work is the lack of consensus on the specific target/mechanism of action of TCP. Although some research suggests a direct impact on the enzymatic activity of TarO (15), other studies suggest that TarO is not the specific target of this molecule (17). Further supporting the notion that TCP does not directly affect TarO is its specificity for NaHCO_3_-responsive MRSA, a strain selectivity not observed with other TarO inhibitors (13, 17). As proposed above, it is possible that TCP also targets the 2’-epimerase Cap5P, a known target of NaHCO_3_ in NaHCO_3_-responsive strains, similar to the ability of tunicamycin to target the 2’-epimerase MnaA (16). Additional investigations are needed to identify the specific targets of both NaHCO_3_ and TCP, as they relate to WTA synthesis, and the mechanism of their ability to specifically sensitize NaHCO_3_-responsive MRSA strains to β-lactams.

Overall, our studies herein provide evidence of the translatability of the NaHCO_3_-responsive phenotype as it relates to the sensitization of MRSA to selected β-lactams *in vivo*. NaHCO_3_ appears to be sensitizing MRSA to β-lactams, in part, via impacts on WTA synthesis. Future development of inhibitors of the WTA pathway holds a promise to “rescue” β-lactam effectiveness for clinical MRSA treatment.

## MATERIALS AND METHODS

### Bacterial isolates and growth conditions

The MRSA isolates utilized in this study are clinical BSI or SSTI isolates that have been previously described and characterized for their NaHCO_3_-responsive phenotypes (4, 6). Isolates were stored at −80°C until thawed for use and isolated on tryptic soy agar (TSA). For all *in vitro* susceptibility assays, cells were grown in cation-adjusted Mueller Hinton Broth (BD, Difco) with 100 mM Tris buffer (CA-MHB Tris, pH 7.3 ± 0.1) ± 44 mM NaHCO_3_ (CA-MHB Tris, 44 mM NaHCO_3_) at 37°C. For *in vivo* studies, cells were grown overnight at 37°C in Brain Heart Infusion (BHI, BBL) with or without 32 µg/mL TCP, as this dose of TCP provided the best synergy with CFX in *in vitro* susceptibility assays. Exposure (“sensitizing”) to TCP did not impact overnight cell density prior to animal infection (Fig. S1).

### MIC and quantitative time-kill assays

MIC and time-kill assays were carried out as previously described (4). Briefly, for MIC assays, cells were grown overnight in the indicated growth medium, then diluted to 5 × 10^5^ CFU/mL into the same medium containing twofold serial dilutions of CFX, with or without 32 µg/mL TCP, on 96-well plates, as per Clinical and Laboratory Standards Institute guidelines (38, 39). Plates were incubated overnight at 37°C, and the MIC was scored at the first well in which turbidity was visually reduced. For time-kill assays, cells were grown overnight in the indicated growth medium, then diluted to 5 × 10^5^ CFU/mL in the same growth medium on 96-well plates and incubated at 37°C for 3 h to reach log phase. After reaching the log phase, the cells were diluted back to 5 × 10^5^ CFU/mL into the same growth medium in the presence or absence of 30 µg/mL CFX and 32 µg/mL TCP. This TCP concentration was selected following extensive piloting studies revealing this to be the optimal concentration to disclose synergy between TCP and CFX *in vitro*. Viable cells were quantified by plating on TSA at 0, 2, 4, and 24 h following incubation with drug.

### *Ex vivo* PK-PD SEV model

Antibiotic PK-PD simulations were performed with *ex vivo* SEVs prepared as previously described (8). SEVs contain 3–3.5 g/dL of albumin and 6.8–7.4 g/dL of total protein (equating to human physiologic levels) and weigh ~0.7 g. The central (“fluid”) compartment model for the SEVs consists of a 150-mL flask glass, maintained at 35–37°C ambient air and fresh media (MHB or MHB + 44 mM NaHCO_3_) instilled via a continuous syringe pump system (New Era Pump Systems, Inc.). The models were performed in duplicate flasks to ensure reproducibility with two SEVs collected for each time point (*n* = 4 SEVs per time point). Simulated human-equivalent antibiotic regimens used for severe infections were derived from human PK data as follows: CFX 750 mg every 8 h, infused over 2 min (C_max_ 50 µg/mL, C_min_ 0.72 µg/mL, T1/2 1.2 h). Standard TCP regimens of 250 mg twice daily in humans display nonlinear pharmacokinetics, with a terminal half-life of repeated dosing of 4–5 d. Therefore, we simulated steady-state concentrations during the terminal half-life (8 µg/mL) (40) by including TCP 8 µg/mL in the model and supplemental media over a 72-h duration to achieve this constant TCP exposure while simulating the CFX elimination rate constant (k_e_ = 0.53 h^−1^). Model SEVs were sampled and processed for CFU enumeration as previously described (8).

### Rabbit model of MRSA IE

To quantify the synergistic impacts of TCP and endogenous HCO_3_ on CFX activity in NaHCO_3_-responsive vs non-responsive strains, a well-characterized rabbit model of indwelling catheter-induced aortic valve IE was used (41). Rabbits were infected intravenously at 48 h after catheter placement with 2 × 10^5^ CFU/animal of the indicated strain; this inoculum represents the ID_95_ for inducing IE as established by extensive pilot experiments for each strain (data not shown). At 24-h post-infection, the animals were randomized into either untreated control groups (sacrificed at this time-point as a therapeutic baseline) or CFX-treated groups (100 mg/kg administered by intramuscular injection, tid for 4 d). This CFX dosing regimen was based on previously established CFX dosing strategies in MSSA rabbit IE models (4, 42, 43).

For CFX-treated animals, at 24 h after the last CFX treatment (to circumvent any antibiotic carryover effects), animals were sacrificed, and their cardiac vegetations, kidneys, and spleen were aseptically removed and quantitatively cultured on TSA plates as previously described (44). For untreated controls, animals were similarly sacrificed at 24-h post-infection to establish a baseline target tissue MRSA bio-burden to compare to CFX-treated animals. Counts were expressed as mean log_10_ CFU per gram of tissue (± SD). The limit of detection in target organ cultures in this model, based on average target tissue weights, is ≤2 log_10_ CFU/g. All culture-negative vegetations, although designated as “sterile,” were still assigned a limit-of-detection log_10_ CFU/g designation based on their weights, for statistical purposes, to enable comparisons between groups. This is a standard procedure used in this model.

### Statistical analyses

An unpaired Student’s *t*-test was used to make all statistical comparisons, with *P < 0.05, **P < 0.01, ***P < 0.001, and ****P < 0.0001. For *in vitro* kill curves, statistics were calculated on the viable cell means at 24 h for each group.

## SUPPLEMENTAL MATERIAL

Figure S1 aac.01627-23-s0001.jpg Impact of ticlopidine pre-exposure on overnight cell density.

ASM does not own the copyrights to Supplemental Material that may be linked to, or accessed through, an article. The authors have granted ASM a non-exclusive, world-wide license to publish the Supplemental Material files. Please contact the corresponding author directly for reuse.
