# Supplementary figures and images for: Sensitizing methicillin-resistant Staphylococcus aureus (MRSA) to cefuroxime: the synergic effect of bicarbonate and the wall teichoic acid inhibitor ticlopidine
Source: Antimicrob Agents Chemother. 2024 Feb 13;68(3):e01627-23. doi: 10.1128/aac.01627-23 (PMC10916381; doi:10.1128/aac.01627-23)

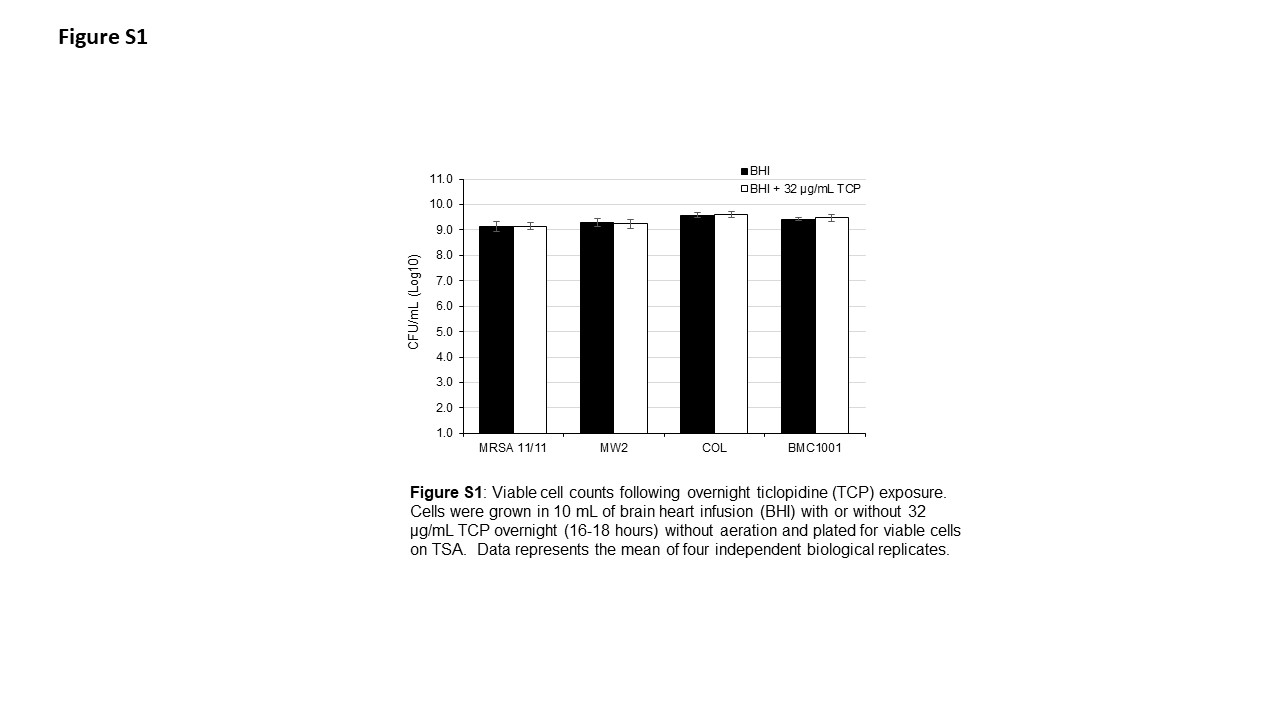

Supplement: Figure S1 — Impact of ticlopidine pre-exposure on overnight cell density. [file aac.01627-23-s0001.jpg]
